# Nudging Social Media toward Accuracy

**DOI:** 10.1177/00027162221092342

**Published:** 2022-05-05

**Authors:** Gordon Pennycook, David G. Rand

**Keywords:** misinformation, nudges, interventions, social media, inattention

## Abstract

A meaningful portion of online misinformation sharing is likely attributable to Internet users failing to consider accuracy when deciding what to share. As a result, simply redirecting attention to the concept of accuracy can increase sharing discernment. Here we discuss the importance of accuracy and describe a limited-attention utility model that is based on a theory about inattention to accuracy on social media. We review research that shows how a simple nudge or prompt that shifts attention to accuracy increases the quality of news that people share (typically by decreasing the sharing of false content), and then discuss outstanding questions relating to accuracy nudges, including the need for more work relating to persistence and habituation as well as the dearth of cross-cultural research on these topics. We also make several recommendations for policy-makers and social media companies for how to implement accuracy nudges.

It’s not the tweets, it’s the retweets that get you in trouble. . . . You see something that looks good and you don’t investigate it.

—Donald Trump (Interview with Barstool Sports, 2020)^1^

A great deal of concern exists about falsehoods spreading on the Internet, often via social media.^2^ Here we review a burgeoning literature on a relatively new approach to the online misinformation problem that has been gaining traction: accuracy “nudges” (or prompts, or primes) that increase the quality of content that users share on social media. We focus on accuracy nudges because other common types of interventions against misinformation, such as debunking/fact checking or educational approaches that teach people to identify misinformation, are reviewed extensively elsewhere (Chan et al. 2017; Kozyreva, Lewandowsky, and Hertwig 2020; van der Linden et al. 2021; Traberg, Roozenbeek, and van der Linden, this volume). Accuracy nudges also have the advantages of being extremely fast to administer and not requiring foreknowledge of which news stories are accurate versus misleading. Furthermore, we focus on the sharing of misinformation on social media because it is a context in which social science–based interventions might actually be implemented by technology companies (unlike, say, focusing on foreign influence operations, domestic political elites, established news organizations, or talk radio). We give an overview of the current state of knowledge and illuminate what we believe to be the most important next steps for investigating accuracy nudge approaches.

## Inattention to Accuracy

A multitude of reasons exist for why one might share false or misleading news on social media. Perhaps the content triggers some sort of moral or emotional response (Brady et al. 2017; Brady, Crockett, and Van Bavel 2020); or it could be reaffirming of one’s political (or other) identity (Van Bavel and Pereira 2018); or it could simply seem amusing, interesting, or important (Chen, Pennycook, and Rand 2021). However, each of these things is not, of course, unique to falsehoods (although in some—perhaps many—cases, inaccurate content may actually be more engaging than accurate content). Thus, rather than asking what causes people to share falsehoods per se, a key question is what factors preferentially cause people to share false news *relative to* true news—that is, what influences the overall quality of content that people share (sometimes referred to as sharing *discernment*; Pennycook and Rand 2021b)?

Undoubtedly, this question has many answers. Here, we focus on one such explanation, namely, a lack of attention to accuracy when deciding what to share on social media (Pennycook et al. 2021). By this account, even if people (1) are able to tell truth from falsehood and (2) have a strong preference not to share inaccurate news (i.e., would not choose to share news they realized to be inaccurate), they might still share misinformation due to *inattention*. This account is premised on the idea that, due to cognitive constraints, attention is limited when people make sharing decisions. The way in which many people use social media—for example, scrolling quickly through a large number of posts, consuming news intermixed with content for which accuracy is not relevant (e.g., family photos, cat videos, etc.), using social media as a way to relax and unwind rather than think critically—is not conducive to thinking carefully about accuracy. Instead, social media focuses users’ attention on factors other than accuracy, such as *social* factors. For example, social media platforms provide immediate, highly quantitative social information about each post (e.g., how many people, and which specific friends, shared and liked each post), which is likely to focus attention on these social cues and has been shown to affect sharing intentions (Avram et al. 2020). Furthermore, the vocal minority of Americans who are highly partisan may consume a disproportionate amount of airtime on social media, which could cause people to focus more on partisanship (and less on accuracy) than they otherwise would. Thus, people who would choose to not share content they realize is inaccurate might never stop to consider the content’s accuracy in the first place and thereby end up sharing misinformation (thus acting against their actual preference).

## Formalizing the Inattention-Based Account of Misinformation Sharing

This inattention account was formalized by Pennycook and colleagues (2021) using a limited-attention utility model. The modeling framework combines three lines of theory. The first is utility theory, which is the cornerstone of economic models of choice (Barberis 2013; Stigler 1950). When people are choosing across a set of options (in our case, whether to share a given piece of content), they preferentially choose the option that gives them more utility, and the utility they gain for a given choice is defined by their *preferences*. In virtually all such models, preferences are assumed to be fixed (or at least to change over much longer timescales than that of any specific decision, e.g., months or years).

The second line of theorizing involves the importance of attention. A core tenet of psychological theory is that when attention is drawn to a particular dimension of the environment (broadly construed), that dimension tends to receive more weight in subsequent decisions (Ajzen 2001; Simon and Newell 1971). While attention has been a primary focus in psychology, it has only recently begun to be integrated with utility theory models—such that attention can increase the weight put on certain preference dimensions over others when making decisions (Bordalo, Gennaioli, and Shleifer 2012; Koszegi and Szeidl 2012).

A third major body of work documents how our cognitive capacities are limited (and our rationality is bounded) such that we are not able to bring all relevant pieces of information to bear on a given decision (Camerer, Loewenstein, and Rabin 2004; Evans and Stanovich 2013; Simon 1972). While the integration of cognitive constraints and utility theory is a core topic in behavioral economics, this approach has typically not been applied to attention and the implementation of preferences. Combining these three approaches, Pennycook et al. (2021) presented a model in which attention operates via cognitive constraints: agents are limited to only considering a subset of their preferences in any given decision, and attention determines which preferences are considered. Importantly, therefore, in this model it is attention—and not preferences (or motivations)—that is affected by context/interventions.

Consider a piece of content *x*, which is defined by *k* different characteristic dimensions; one of these dimensions is whether the content is false/misleading *F*(*x*), and the other *k*-1 dimensions are non–accuracy related (e.g., partisan alignment, humorousness, etc.) defined as *C_2_*(*x*) . . . *C_k_*(*x*). In our model, the utility a given person expects to derive from sharing content *x* is given by

$$U(x)=-a_{1}\beta_{F}F(x)+\sum_{i=2}^{k}a_{i}\beta_{i}C_{i}(x)$$

where *β_F_* indicates how much they dislike sharing misleading content and *β_2_* . . . *β_k_* indicate how much they care about each of the other dimensions (i.e., *β*s indicate preferences). Meanwhile, *a_1_* indicates how much the person is paying attention to accuracy, and *a_2_* . . . *a_k_* indicate how much the person is paying attention to each of the other dimensions. The probability that the person chooses to share the piece of content *x* is then some increasing function of *U*(*x*).

In the standard utility theory model, *a_i_* = 1 for all *i* (all preferences are considered in every decision). Thus, preference terms with larger *β* values necessarily exert more influence on sharing decisions. The limited-attention model of Pennycook et al. (2021) instead uses the *a_i_* values to incorporate cognitive constraints: people can consider only a subset of characteristic dimensions when making decisions. Specifically, agents can only attend to *m* out of the *k* utility terms in a given decision. That is, each value of *a* is either 0 or 1, *a_i_*∊{0,1}; and because only *m* terms can be considered at once, the *a* values must sum to *k*. Critically, the probability that any specific set of preference terms is attended to (i.e., which *a* values are equal to 1) is heavily influenced by the situation, and (unlike preferences) can change from moment to moment.

Thus, even if people have a strong preference for not sharing inaccurate content (i.e., *β_F_* is as large, or larger than, other *β* values), how accurate content is may still have little impact on what people decide to share *if the context focuses their limited attention on other dimensions*. The accuracy-based account of misinformation sharing, then, is the hypothesis that *β_F_* is not smaller than the other *β* values (e.g., the *β* for political concordance, such that people do not want to share false but politically concordant news), but that people nonetheless sometimes share misinformation because the probability of observing *a_1_* = 1 is far less than 1 (*p*[*a_1_* = 1] << 1)—that is, people often fail to consider accuracy. That is, this account predicts a substantial disconnect between accuracy judgments and sharing intentions, whereby accuracy judgments will be much more sensitive to the veracity of news content than sharing intentions will be. Furthermore, the inattention-based account predicts that prompts that cause people to attend to accuracy can increase veracity’s role in sharing by increasing the probability that *a_1_* = 1 (*p*[*a_1_* = 1] | treatment > *p*[*a_1_* = 1] | control). That is, the accuracy prompt shines an attentional “spotlight” on the accuracy dimension, increasing its chance to influence judgments. Importantly, our theory proposes that accuracy prompts lead people to reallocate their attention to different preferences (i.e., the *a* terms) and do *not* cause changes in one’s preferences/processing goal (which are captured by the *β* terms in the model).

## Disconnect between Accuracy Judgments and Sharing Intentions

In support of the model’s first prediction, experiments have revealed a strong disconnect between accuracy judgments and sharing intentions in survey experiments. (One cannot easily assess accuracy judgments in observational studies using social media data, making survey experiments a key tool for investigating these questions.) In these experiments, participants are randomized to either rate the accuracy of a series of news posts or indicate their willingness to share the same series of news posts. When people are asked to judge accuracy (and thus have their attention focused on accuracy), they are often very good at distinguishing between true and false news headlines (e.g., averaging the accuracy ratings of even small groups of laypeople can produce as much agreement with professional fact-checkers as professional fact-checkers show with each other; Allen et al. 2021).

However, even in these situations in which people who are asked about accuracy can identify news veracity very well, veracity typically has very little impact when people are asked whether they would share the headlines online (sans an accuracy prompt) (Epstein et al. 2021; Pennycook et al. 2021; Pennycook, McPhetres, et al. 2020). Instead, whether the news aligns with the respondents’ partisanship is a much stronger predictor of sharing than whether the news is true. This stands in stark contrast to accuracy judgments, where truth is a far stronger predictor than partisan alignment. Thus, a common finding is that the fraction of false headlines selected for sharing is meaningfully larger than the fraction of false headlines that are rated as accurate—implying that people are willing to share headlines that they would be able to identify as being accurate if they thought about it (see also Fazio 2020).

## Using Accuracy Prompts to Improve Sharing Discernment

The inattention account posits that this disconnect is due to people forgetting or neglecting to consider accuracy in their sharing decisions. Therefore, shifting people’s attention toward the concept of accuracy will cause them to be more discerning in their sharing. An alternative account for this pattern, however, is that people knowingly share false content (e.g., because they are trolling or have a need for chaos; Arceneaux et al. 2021), in which case shifting people’s attention toward the concept of accuracy will not have a meaningful impact since they already realize the content is inaccurate and are choosing to share it anyway.

These competing accounts can be disambiguated using experiments that prime the concept of accuracy. The results of a series of such experiments support the inattention account. For example, if participants are asked to rate the accuracy of a single politically neutral news headline at the outset of a survey experiment (an “Evaluation” accuracy prompt treatment, sometimes introduced to participants as part of a pretest for a different study), their subsequent sharing of news relating to politics or COVID-19 (Epstein et al. 2021; Pennycook et al. 2021; Pennycook, McPhetres, et al. 2020; Roozenbeek, Freeman, and van der Linden 2021) becomes more discerning (e.g., people share relatively less false content compared to a control condition where attention is not drawn to accuracy). Fitting the limited-attention utility model to data from such experiments finds that, as per the inattention-based account, the average participant has a strong preference to not share inaccurate news (the *β* on not sharing inaccurate content was as large or larger than the *β* value for sharing politically concordant news), but participants often failed to attend to accuracy (*a* value on the accuracy-related preference term was 0), leading them to indicate that they would share inaccurate news. Supporting the model’s interpretation that the accuracy nudge works by redirecting attention rather than altering motivations/preferences, Pennycook et al. (2021) found that the treatment had no significant effect on responses to a postexperimental question asking, “How important is it to you that you only share news articles on social media (such as Facebook and Twitter) if they are accurate?”

Critically, the accuracy prompt effect is not driven simply by asking participants any question at the beginning of the experiment: asking about how funny or entertaining the headlines were had no impact on subsequent sharing (i.e., the extent to which people share more true content than false content) (Pennycook et al. 2021). Furthermore, the Evaluation intervention is not the only way to shift attention toward accuracy. Research has used several different types of accuracy prompts, providing support for strong conceptual replicability (Epstein et al. 2021; Pennycook and Rand 2021c). For example, providing minimal digital literacy tips (Epstein et al. 2021) or simply asking participants how important it was to them to share only accurate news (Pennycook et al. 2021; Epstein et al. 2021) increased sharing discernment. Epstein et al. (2021) also found that the effect was particularly strong (i.e., a 100 percent increase in sharing discernment) for a more lengthy intervention where participants judged the accuracy of four headlines and were told whether each response was correct to reinforce attention to accuracy. In contrast, simply providing descriptive norm information by telling participants that *other people* thought it was important to share only accurate news was not particularly effective—perhaps because such interventions also draw attention to social considerations and not just accuracy.

How robust is this accuracy prompt effect? In a recent meta-analysis, we analyzed every experiment we ran (*k* = 20, *N* = 26,863) with accuracy prompts in U.S. samples between 2017 and 2020 (Pennycook and Rand 2021c). Typical meta-analyses have two major drawbacks that this approach overcomes: publication bias and questionable research practices (and, in particular, p-hacking) (Carter et al. 2019). With respect to publication bias, since our meta-analysis includes the entire “file drawer,” inclusion is not conditioned on the outcome of the study and, hence, no publication bias exists. Standard meta-analyses cannot guarantee that publication bias has not occurred because it is not possible to know if all possible experiments have been included (Rothstein, Sutton, and Borenstein 2006). With respect to p-hacking, our meta-analysis uses the exact same analytic procedure across all experiments, and this procedure was preregistered in the earliest experiments. Thus, the effect size estimates in individual studies are not influenced by researcher degrees of freedom (Simmons, Nelson, and Simonsohn 2011). Consequently, the effect size estimate produced by the meta-analysis is more credible than typical meta-analyses. We also note that although our meta-analysis only represents the studies from a single research group, we believe that less is gained by independent replications relative to physical lab studies because all studies were run online; it is not clear how much is gained from having an identical study launched by a different group.

The overall effect of accuracy prompts across the twenty experiments was a 72 percent increase in sharing discernment relative to control. The effect was stronger for longer interventions and was not significantly moderated by gender, race, political ideology, education, or the value people explicitly placed on accuracy. (Some evidence existed of moderation by political partisanship, such that accuracy prompts were less effective among Republicans despite having an overall positive effect; however, this moderation only occurred robustly for convenience samples recruited from Amazon’s Mechanical Turk and not for more representative samples from Lucid and YouGov.) We also found that the effect was larger for people who are older, who are higher in cognitive reflection, and who passed more attention check questions (although the effect was observed even among younger adults and those low in cognitive reflection). The central conclusion from these analyses is that, although some heterogeneity occurred in the size of the effect, it is quite robust across subgroups. Nonetheless, we do not know how effective the intervention would be against so-called super-spreaders (Grinberg et al. 2019; Guess, Nyhan, and Reifler 2020), who share a very large amount of misinformation on social media, since we cannot tell whether these people are represented in our surveys.

One of the key pieces of evidence that accuracy prompts improve sharing discernment by drawing attention to accuracy is that the effect is calibrated to the underlying plausibility of the headlines. That is, research has consistently shown that the treatment effect is most strongly negative for headlines that are judged to be the most implausible (based on out-of-sample ratings of headline likelihood) (Epstein et al. 2021; Pennycook et al. 2021; Pennycook, McPhetres, et al. 2020; Roozenbeek, Freeman, and van der Linden 2021). The meta-analysis found a correlation of *r* = –.757 between headline plausibility and the treatment effect across fifteen experiments, thus providing strong evidence that accuracy prompts reduce sharing to the extent that headlines are likely to be perceived as inaccurate. This makes sense, given the proposed attentional mechanism: an increased focus on accuracy can only impact sharing insofar as headlines are readily discernable as false (or true).

Do accuracy prompt effects generalize from survey experiments to actual behavior on social media? To shed light on this question, Pennycook et al. (2021) conducted a digital field experiment (for a review of this methodology, see Mosleh, Pennycook, and Rand 2021). Specifically, Twitter users who had (re)tweeted articles from Breitbart and Infowars (two highly partisan right-wing websites that produce misleading content) were followed by one of several innocuous “bots” (e.g., “CookingBot”). The users who followed back the bot accounts formed the subject pool of the experiment and were sent a private message asking them to judge the accuracy of a neutral news headline (replicating the Evaluation intervention from the survey experiments). To allow causal inference, the study used a stepped-wedge design in which all users received the message but were randomly assigned to treatment date. Naturally, very few users responded to the message, but it is not necessary for them to respond—simply reading the message is sufficient to be “treated.” Accordingly, intent-to-treat analyses found that a significant increase occurred in the quality of news content that users shared in the 24 hours following the direct message, as measured by the trustworthiness of the news sources that were shared. In addition to demonstrating the practical applicability of the accuracy nudge approach, this field experiment, in which participants did not know they were part of a study, helps to address potential concerns about experimental demand effects/social desirability in the survey experiments. (Also relevant is the observation from the 2021 survey experiment of Epstein and colleagues (2021) that a descriptive social norms treatment—which is likely to invoke social desirability more than the Evaluation treatment—had no effect on sharing discernment.)

## Outstanding Questions and Future Directions

Although clear evidence exists in both survey experiments and a digital field experiment that accuracy prompts can improve the quality of content that people share on social media, nonetheless various outstanding questions remain about the approach that need to be addressed. Most fundamentally, work on accuracy prompts has focused almost entirely on the United States, despite the global nature of online misinformation. Cross-cultural studies are essential for assessing the generalizability of the approach. Fortunately, some early evidence indicates that accuracy prompts are broadly effective across cultures (Arechar et al. 2022).

A key practical question relates to the time course of the effect: how quickly does the effect wear off? It is difficult to evaluate this question in survey experiments. Although the meta-analysis of Pennycook and Rand (2021c) does not find evidence of the effect decaying over time, experimental sessions are typically not very long. The digital field experiment on Twitter that was mentioned earlier recorded tweets in the 24 hours that followed the intervention, but the experiment did not have sufficient power to test for longer effects; and we could not possibly observe when the message was read, making it even harder to assess persistence. From a theoretical perspective, given the fickle nature of attention, we have little reason to believe that the interventions would last particularly long. Thus, rather than a one-time treatment to reduce misinformation, the implication of the accuracy prompt studies is that attention matters—and that, therefore, technology companies need to make fundamental changes to the social media ecosystem to systematically redirect attention to accuracy.

Another factor to consider is whether people would become habituated to accuracy prompts, thereby decreasing the impact of the intervention in the long run. A natural solution to this problem is for platforms to use a wide variety of accuracy nudges, constantly rotating which prompts are applied for any given user. Such variation should help to mitigate habituation. Accuracy prompts may also cause people to adopt the habit of pausing and thinking about accuracy before they share content on social media, even absent any reminders. Ultimately, future research should determine the potential downstream consequences of a long-term accuracy prompt campaign. This means that social media companies need to think carefully about, and run experiments to determine, optimal implementation rather than simply deploying the designs used in our studies. Ideally, the results of these experiments would be shared with the public, including investigating potential negative externalities (such as decreasing sharing of accurate news from unknown, and thus distrusted, sources; Pennycook and Rand 2019b).

Another important practical question is how accuracy prompts may interact with other approaches to fighting misinformation. One appeal of accuracy prompts relative to most other interventions is that accuracy prompts entirely preserve user autonomy and do not require any centralized definition of truth versus falsehood. Nonetheless, accuracy prompts may interact beneficially with more traditional approaches. One clear point of synergy involves attempts to leverage crowdsourcing to identify misinformation at scale (Allen et al. 2021; Epstein, Pennycook, and Rand 2020; Pennycook and Rand 2019a). Periodically asking users to rate the accuracy of content while they scroll through their newsfeed will not only prompt them to consider accuracy when making subsequent sharing decisions but can also help to generate crowd ratings that platforms can use to help inform ranking algorithms or misinformation labeling. Accuracy prompts are also likely to work in a synergistic fashion with media literacy and other educational or fact-checking interventions aimed at improving users’ truth discernment (Guess et al. 2020; van der Linden et al. 2021; Nieminen and Rapeli 2018; Traberg, Roozenbeek, and van der Linden, this volume). An increased focus on accuracy can only improve sharing insofar as users can successfully assess which headlines are inaccurate, and so improving users’ truth discernment will magnify the impact of accuracy prompts. Conversely, increasing users’ understanding of what is accurate or inaccurate will not have a strong impact on sharing if they fail to consider accuracy when deciding what to share. Thus, accuracy prompts will magnify the long-term impact of media literacy interventions on subsequent sharing.

One might expect warnings or corrections for misinformation, either from fact-checkers or from other users, to also work as accuracy prompts. However, because they are often only applied to a small fraction of inaccurate content (due to scalability issues), fact-checker warnings may unintentionally boost belief in, and sharing of, unlabeled false claims if users infer that the absence of a tag implies verification (the “implied truth effect”) (Pennycook, Bear, et al. 2020). And a recent field experiment on social corrections delivered by bots designed to look like other users found that social corrections decreased the quality of news that users subsequently retweeted (Mosleh, Martel, et al. 2021)—the opposite of what we found using the Evaluation accuracy nudge intervention. Future work should identify what messaging features most effectively redirect attention to accuracy without causing reactance or distraction.

Researchers should explore theoretical issues relating to inattention to accuracy and the efficacy of accuracy prompts. For example, if people are not paying attention to accuracy when making judgments about what to share, what *are* they paying attention to? It seems likely that the social signaling and feedback mechanisms that are inherent to most social media applications (such as “likes”) reinforce social considerations, but what precisely are these considerations? Relatedly, content that is highly emotional (Martel, Pennycook, and Rand 2020) or that contains moral language (Brady, Crockett, and Van Bavel 2020) will likely draw people’s attention and perhaps undermine their ability, or willingness, to pause and consider the accuracy of the content. Recent work also shows that news headlines that seem to be important or timely are more likely to be shared (and that, with a set of close to two hundred headlines, false headlines seemed more important) (Chen, Pennycook, and Rand 2021). Future work should investigate these issues in more detail.

Another theoretical issue pertains to the role of intuitive versus analytic thinking in the sharing of misinformation and how this intersects with inattention to accuracy. Specifically, research has shown repeatedly that people who are more reflective and analytic (relative to those who tend to rely on intuitive gut feelings) are better able to distinguish between true and false news (Pennycook and Rand 2019b, 2019c) (for a meta-analysis, see Pennycook and Rand 2021a) and that people are more likely to believe false news (but not true news) when they are forced to rely on their intuition (Bago, Rand, and Pennycook 2020) or are instructed to rely on emotion (Martel, Pennycook, and Rand 2020). Furthermore, evidence exists that people who are more reflective are also more discerning in what they share on social media, both in survey studies (Pennycook and Rand 2021c) and on Twitter (Mosleh, Pennycook, et al. 2021) (although see Osmundsen et al. [2021], who find no relationship between cognitive reflection and fake news sharing on Twitter). Furthermore, people who are more reflective are also more strongly impacted by the accuracy prompt intervention (as noted above) (Epstein et al. 2021; Pennycook and Rand 2021c). It seems plausible, then, that part of the reason why people are inattentive to accuracy is because they are not engaging in sufficient reflection on social media. Alternatively, however, accuracy prompts may not induce more reflection but merely change what factors people are reflecting about (Lin, Pennycook, and Rand 2022). Future empirical and theoretical work should distinguish between these possibilities.

## Conclusion

We argue that at least some sharing of misinformation on social media can be attributed to users merely failing to consider accuracy when deciding what to share. We have outlined a limited-attention utility model that formalizes our theoretical perspective on this issue. Consistent with this model, experimental evidence consistently indicates that simply redirecting attention to the concept of accuracy can increase sharing discernment. Nonetheless, several outstanding questions remain relating to accuracy nudges that we surfaced here. We hope that this current review will serve as a useful guide for future research, as well as for policy-makers and social media companies who may wish to implement accuracy nudges.

## Notes

1. See https://www.independent.co.uk/news/world/americas/us-politics/donald-trump-twitter-tweets-barstool-interview-dave-portney-a9637186.html.

2. Researchers may disagree on the scope of the problem, a debate we will not rehash here.
